# Renal Benefits of SGLT 2 Inhibitors and GLP-1 Receptor Agonists: Evidence Supporting a Paradigm Shift in the Medical Management of Type 2 Diabetes

**DOI:** 10.3390/ijms20235831

**Published:** 2019-11-20

**Authors:** Vjera Ninčević, Tea Omanović Kolarić, Hrvoje Roguljić, Tomislav Kizivat, Martina Smolić, Ines Bilić Ćurčić

**Affiliations:** 1 Department of Pharmacology, Faculty of Medicine, Josip Juraj Strossmayer University of Osijek, J. Huttlera 4, 31000 Osijek, Croatia; vnincevic@mefos.hr (V.N.); tomanovic@mefos.hr (T.O.K.); hrvoje.roguljic@mefos.hr (H.R.);; 2 Department of Pharmacology and Biochemistry, Faculty of Dental Medicine and Health, Josip Juraj Strossmayer University of Osijek, Crkvena 21, 31000 Osijek, Croatia; 3 Department for Cardiovascular Disease, University Hospital Osijek, 4, 31000 Osijek, Croatia; 4 Clinical Institute of Nuclear Medicine and Radiation Protection, University Hospital Osijek, 31000 Osijek, Croatia; tkizivat@mefos.hr; 5 Department for Nuclear Medicine and Oncology, Faculty of Medicine, Josip Juraj Strossmayer University of Osijek; J. Huttlera 4, 31000 Osijek, Croatia; 6 Department of Diabetes, Endocrinology and Metabolism Disorders, University Hospital Osijek, 31000 Osijek, Croatia

**Keywords:** diabetic nephropathy, diabetes mellitus, GLP-1 receptor agonists, SGLT2 inhibitors, molecular mechanisms

## Abstract

Diabetic nephropathy (DN) is one of the most perilous side effects of diabetes mellitus type 1 and type 2 (T1DM and T2DM).). It is known that sodium/glucose cotransporter 2 inhibitors (SGLT 2i) and glucagone like peptide-1 receptor agonists (GLP-1 RAs) have renoprotective effects, but the molecular mechanisms are still unknown. In clinical trials GLP-1 analogs exerted important impact on renal composite outcomes, primarily on macroalbuminuria, possibly through suppression of inflammation-related pathways, however enhancement of natriuresis and diuresis is also one of possible mechanisms of nephroprotection. Dapagliflozin, canagliflozin, and empagliflozin are SGLT2i drugs, useful in reducing hyperglycemia and in their potential renoprotective mechanisms, which include blood pressure control, body weight loss, intraglomerular pressure reduction, and a decrease in urinary proximal tubular injury biomarkers. In this review we have discussed the potential synergistic and/or additive effects of GLP 1 RA and SGLT2 inhibitors on the primary onset and progression of kidney disease, and the potential implications on current guidelines of diabetes type 2 management.

## 1. Introduction

Diabetic nephropathy (DN) is a complication of diabetes mellitus, both type I and II, caused by changes in microvasculature [1], and which can lead to end-stage renal disease and cardiovascular disease [2,3]. Moreover, it is the leading cause of chronic kidney disease, affecting 30–40% of patients with diabetes mellitus type 1 and 25–40% of patients with diabetes type 2 [4,5,6,7].

In its classic definition DN is defined as increased protein excretion in urine [8]. Major representations of diabetes in renal disease include persistent albuminuria, loss of podocytes, glomerular hypertrophy, matrix expansion, and thickening of the glomerular basement membrane [9,10]. The first stages of DN are characterized by microalbuminuria, a small increase in albumin excretion in urine [11,12,13]. Later stages are defined by macroalbuminuria or proteinuria leading to a decreased glomerular filtration rate [8].

Pathogenesis and progression of diabetic kidney disease are most likely a result of interactions between metabolic and hemodynamic changes which are caused by onset of diabetes [14], but also other factors including genetic predisposition [15], generation of reactive oxygen species (ROS) caused by hyperglycemia [14,15,16], and inflammation [17].

Studies have shown that diabetic kidney disease can lead to end stage renal disease in 30–40% of diabetes mellitus (DM) patients [18], implying that genetic variations could have impact on the start and progression of DM and the end stage renal disease. Genome-wide studies have been conducted to identify potential candidate genes of importance to DM and diabetic kidney disease [19]. More than fourteen genes have been identified as important to the development of diabetic kidney disease, among which are genes controlling lipid metabolism (*ADIPOQ*), glucose metabolism (*GCKR*), angiogenesis (*EPO promotor* gene), genes related to renal structure and function (*SHROOM3*), inflammation and oxidative stress related genes (*TGF-β1*), renin-angiotensin-aldosterone system related genes (*AGTR1*), and others [15]. Still, the complete effects of these genes and their variants are not completely clear. Results from these studies cannot be replicated among different races indicating race-specific gene polymorphisms or still significant environmental impacts.

Inflammation and chronic high levels of circulating glucose and its end metabolites are main causes of tissue damage in DM by, among other, creating high levels of oxidative and nitrosative stress in kidneys [20,21]. The effects of oxidative stress in other renal diseases like urolithiasis have been described [22]. High production of ROS and nitrosative species (NS) can cause damage to nuclear and mitochondrial DNA, induce apoptosis, and cause endoplasmic reticulum stress, and with this playing a role in cell death pathways such as apoptosis and necrosis in key cell types such as podocytes [20].

Considering the structural changes in diabetic kidney disease, they are similar in both DM1 and DM2, but are more heterogeneous and less predictable in association with clinical presentation in DM2 [23], probably because DM2 has unreliable onset timing, longer exposure to hyperglycemia before diagnosis, older patients, and patients that are treated with renin-angiotensin inhibitors before the onset of diabetes [24].

These structural alterations encompass modifications of several kidney departments. One of the first changes is thickening of the glomerular basal membrane, becoming apparent at 1.5 to 2 years from the diagnosis of DM, and which is closely followed by thickening of the capillary and tubular basement membrane [25,26,27,28]. Glomerular changes later include mesangial matrix expansion, loss of endothelial fenestrations, and loss of podocytes with effacement of foot processes [25]. The first signs of mesangial volume expansion are seen after 5–7 years of DM1 onset [27,28,29,30]. As DM progresses, segmental mesangiolysis appears and is considered to be connected with the development of micoaneurysms and Kimmelstal–Wilson nodules [31,32]. Subendothelial deposits of proteins forming periodic acid-Schiff-positive and electron-dense deposits accumulate in small arterioles, glomerular capillaries and microaneurysms result in exudative lesions and can cause luminal compromise [25]. Subepithelial deposits similar to subendothelial can be seen in Bowman’s capsule and renal tubules. In the later stages of DM, glomerulopathy and interstitial changes grow together to segmental and global sclerosis [25]. Glomerular filtration, albuminuria, and hypertension in DM1 are strongly correlated with mesangial expansion, but to a less degree with glomerular basement membrane width [25].

## 2. GLP-1 Agonists in Diabetic Nephropathy

### 2.1. Classes of GLP-1 RA and Mechanism of Action

Glucagon-like peptide-1 (GLP-1) receptor agonists (RAs), an antidiabetic class of drugs, are known for their proven efficacy and safety profile [33]. They can be divided into human originated GLP-1 RAs, synthesized by various modifications of human GLP-1 active fragments, and agents derived from reptile Gila monster venom (exendin-4) [33,34]. Another classification of GLP-1 RAs is based on their pharmacokinetic profile, which divides these drugs into two different groups: short-acting (exenatide twice daily and lixisenatide) and long-acting agonists (once-weekly injected; dulaglutide, liraglutide, semaglutide, albiglutide) [33,34]. The half-lives of GLP-RAs vary between 2–3 h for short-acting agonists and 13 h to 7 days for long-acting agonists [33]. Up-to-date, above-mentioned 6 GLP-1 RAs have been approved for the treatment of patients with type 2 diabetes mellitus (T2DM), and all these drugs are administered by subcutaneous injection [33]. In general, GLP-1 RAs exert various beneficial effects in T2DM: enhancement of glucose-dependent insulin secretion, acceleration of β-cells proliferation and inhibition of β-cells apoptosis, inhibition of motility and gastric emptying, and a stimulation of the sensations of satiety and fullness by direct action on the central nervous system, with reduction in body weight [34,35,36,37]. Many other effects are still being investigated, among them reduction in systolic and diastolic blood pressure and improvements in lipid profile [33,38,39,40,41,42]. The main difference between the two groups is the fact that short-acting GLP-1 RAs while delaying gastric emptying, mostly lower postprandial plasma glucose, whereas the long-acting agonists predominantly exhibit insulinotropic and glucagonostatic actions, consequently exerting a much greater effect on fasting glucose concentrations. Various meta-analysis showed that long-acting agonists were more successful in lowering HbA_1C_ compering to short-acting agonists [33,41,43,44,45,46,47]. More specifically, these studies demonstrated that the largest reduction in HbA_1c_ values was associated with dulaglutide and exenatide once weekly, whereas the smallest mean reduction was observed with albiglutide [48]. The same results were observed when comparing the effects on the reduction of body weight [33]. The advantages of liraglutide in comparison with exenatide were: less frequent nausea and vomiting (shorter duration of GI side effects at the beginning of the therapy), more efficacy in lowering glycemic parameters, fasting glucose, and improving the homeostatic model assessment of β cells [34]. Generally, the most often side effects of GLP-1 RA therapy are gastrointestinal events (nausea, vomiting, diarrhea; shorter duration in short-acting agonists because of their lack of substantial effects on gastric emptying), and injection-site reactions (notable for albiglutide and lixisenatide), immunogenicity (exendin-4 derivates are more likely to be associated with the development of antidrug antibodies compared to GLP-1 RAs modified from human GLP-1) [33,49,50]. Nevertheless, these adverse events are rarely serious or persistent, nor the cause of discontinuation of therapy, especially because of the high efficiency of these drugs.

### 2.2. Potential Nephroprotective Actions of GLP-1 Agonists

Among all the above-mentioned therapeutic effects (lowering glucose, reducing body-weight etc.), GLP-1 RAs exert possible nephroprotective effects in T2DM, which have been demonstrated in various studies. Yin W et al. showed that GLP-1 RAs reduced albuminuria and ameliorated kidney tubules and tubulointerstitial lesions in the diabetic nephropathy rats model [51]. GLP-1 RAs downregulated the expression of tubulointerstitial tumor necrosis factor alpha (TNFα), monocyte chemoattractant protein-1(MCP-1), collagen I, alpha-smooth muscle actin (α-SMA), and fibronectin (FN) which are all reported to play a role in the diabetic nephropathy [51]. Additionally, the level of C-peptide, which was found to inhibit tubulointerstitial fibrosis [52], was increased by GLP-1 RAs and this may be one of the ways of improving tubuluinterstitial and tubular injury in GK rats with diabetic nephropathy [51]. In the study of Kodera et al. various beneficial effects of exendin-4 were showed, with emphasis on the prevention of macrophage infiltration, decrease of protein levels of intercellular adhesion molecul-1 (ICAM-1) and type IV collagen in glomeruli, as well as the decrease of oxidative stress (downregulation of *Nox4* gene expression) and nuclear factor-kB (known for contributing to cross-talk between inflammation and oxidative stress) activation in kidney tissue [53]. Furthermore, liraglutide is capable of inhibiting NAD(P)H oxidase through generation of cAMP, followed by activation of PKA or Epac2 [54,55,56,57]. Hendarto et al. confirmed the role of liraglutide in the normalization of oxidative stress markers and expression of renal NAD(P)H oxidase components (Nox4, gp91phox, p22phox, p47phox) in diabetic rats, but independently of lowering plasma glucose levels [58]. Similar results were demonstrated in the mouse model of diabetic nephrophathy with the crucial role of liraglutide in protection against renal oxidative stress and lowering of fibronectin accumulation in glomerular capillary walls [59]. Molecular mechanisms included in these actions are inhibition of NAD(P)H oxidase and activation of cAMP-PKA pathway as already explained [59]. The in vitro beneficial effects of liraglutide were also showed in various studies. Zhao et al. proved that liraglutide enhances cell viability in HK-2 cells (human proximal tubular cells) by downregulating caspase-3 expression [37]. Furthermore, mRNA and protein expression of GLP-1R was significantly enhanced by liraglutide, whereas the expression of the autophagic markers LC3-II and Beclin1 was ameliorated [37]. All these effects were blocked by the GLP-1R antagonist exendin-(9–39) [37]. Additionally, another study on HK2 cells treated with GLP1 RAs showed decrease in the expression of profibrotic factors like fibronectin, α-SMA, collagen I, and TNFα [51]. In the same study GLP-1RAs inhibited the activity of NF-κB and p38MAPK (two significant signaling pathways for kidney fibrosis) via GLP-1R [51]. Various studies confirmed the role of GLP-1RAs in water and electrolyte balance. One of the suggested mechanisms for this effect is inhibition of intestinal sodium–hydrogen exchanger isoform 3 (NHE3) activity [60]. This NHE3 exchanger is located on the renal proximal tubule, and GLP-1RA, by inhibiting its activity, enhance natriuresis and diuresis [61]. Accordingly, when adding GLP-1R blocker exendin-9, a decrease in renal excretion of sodium and water is observed [62]. Furthermore, exendin-9 has been connected with slight decrease in glomerular filtration rate (GFR), although it would be expected to increase GFR by increasing proximal tubular reabsorption, followed by inhibition of tubuloglomerular feedback signals and reduction in afferent arteriolar resistance [62]. However, this implicates another possible positive effect of GLP-1RA on nephroprotection and water/sodium balance [62]. Glomerular hyperfiltration enhanced by GLP-1RAs increases filtration and in the end excretion of electrolytes [61]. Finally, all these studies, which show the beneficial effects of GLP-1RAs in diabetic glomerular, tubulointerstitial, and tubular nephropathy, implicate the possible clinical use of these agents in treatment of diabetic nephropathy.

### 2.3. Assessment of Nephroprotective Effect of GLP-1 Receptor Agonists in Clinical Trials

Recent clinical trials demonstrate notable evidence of glucagon-like peptide-1 (GLP-1) agonists exerting renal benefits.

Between June 2012 and August 2013 the LIRA–RENAL trial examined the efficacy and safety profile of liraglutide in diabetic patients with moderate renal impairment (defined as eGFR 30–59 mL/min/1.73 m^2^) [63]. This double blinded, randomized, placebo-controlled trial included 279 patients with type 2 DM who had HbA1c in the range of 7% to 10%. Addition of liraglutide to background glucose-lowering therapy reduced HbA1c more than placebo treatment (−1.05% vs. −0.38%). During the trial no deterioration of renal function was observed in patients treated with liraglutide in comparison with placebo. Furthermore, albuminuria assessed as the urinary albumin-to-creatinine ratio showed lower increase at week 26 in patients treated with liraglutide, although it was not significantly.

A more extensive and longer study of liraglutide treatment effect on renal outcomes in patients with diabetic nephropathy was the Liraglutide Effect and Action in Diabetes: Evaluation of Cardiovascular Outcome Results (LEADER) trial [64]. The LEADER trial included 9340 patients with type 2 diabetes and a high risk of cardiovascular disease with a median follow-up of 3.84 years. 23.1% of the trial population had mean estimated GFR less than 59 mL per minute per 1.73 m^2^; and furthermore, microalbuminuria and macroalbuminuria were present at the baseline (26.3% and 10.5%, respectively). The renal outcome showed a lower rate of occurrence in the liraglutide group in comparison with placebo (5.7% vs. 7.2%). That was primarily the result of lower incidence of new-onset persistent macroalbuminuria in patients treated with liraglutide (3.4% vs. 4.6%). During the follow-up the urinary albumin-to-creatinine ratio growth was slower in liraglutide group while decline of estimated GFR and the rates of renal adverse effects were similar between the two groups. Interestingly, the effect of liraglutide on composite renal outcomes showed no difference in prespecified subgroups of patients with an elevated baseline renal risk, suggesting effectiveness of the liraglutide renal benefit to be an independent factor of stage of chronic kidney disease.

While the LEADER trial included patients with macroalbuminuria from the baseline to the onset of sustained albuminuria in the endpoint, the Evaluation of Lixisenatide in Acute Coronary Syndrome (ELIXA) trial assessed renal outcomes in patients only with normoalbuminuria or microalbuminuria [65]. This randomized, double blinded study primarily examined the effect on cardiovascular outcomes of short-acting GLP1 agonist lixisenatide in 6068 patients with type 2 diabetes and a recent acute coronary syndrome. Beside cardiovascular safety, the addition of lixisenatide to standard therapy showed beneficial impact on renal outcomes. The renoprotective effect manifested as lower rate of increase in urinary albumin-to-creatinine ratio, 34% in the placebo group vs. 24% in lixisenatide group. No significant difference was observed between the two groups in eGFR decline or doubling of serum creatinine, while overall incidence of renal adverse effects was low in both groups. Evidently the difference in pharmacokinetics of GLP-1 analogs, short-acting versus long-acting analogs, has no impact on renal composite outcomes. Somewhat different results were obtained in AWARD-7 study which examined the effect of long acting GLP-1 analog dulaglutide versus insulin glargine on renal outcomes in patients with diabetes type 2 and moderate-to-severe CKD [66]. 577 participants were randomized in three groups, dulaglutide 1.5 mg or 0.75 mg treated groups and insulin glargine group. The decline of eGFR after 52 weeks of treatment with dulaglutide was significantly smaller in dulaglutide treated patients compared to insulin group. The albuminuria decrease was similar between all treatment groups, with a slightly greater decrease of urinary albumin-to-creatinine ratio in dulaglutide treated patients. Although dulaglutide enhanced body loss, whether fat or muscle tissue, the study confirmed the beneficial renal effect of GLP-1 analog as an independent action. In the Trial to Evaluate Cardiovascular and other Long-term Outcomes with Semaglutide in Subjects with Type 2 Diabetes (SUSTAIN-6), the rate of persistent macroalbuminuria was lower in patients receiving semaglutide, than in those receiving placebo [67].

Taken together, GLP-1 analogs as antidiabetic medications show significant influence on renal composite outcomes, primarily on a new onset of macroalbuminuria as shown in Table 1. Numerous previous studies characterized development of albuminuria as an independent predictor of diabetic nephropathy progression with subsequent deterioration of estimated glomerular filtration and development of end stage renal disease [68,69,70]. Definitely, more intensive glycemic control achieved by addition of GLP-1 agonist to background antidiabetic therapy justifies a decrease of macroalbuminuria incidence in diabetic patients due to a well-known effect of serum high glucose concentration on increased filtration rate of proteins via glomerular capillary membrane and on impaired tubular reabsorption [71]. Another possible mechanism of GLP-1 beneficial renal effect could be suppression of inflammation-related pathways. Preclinical studies have unambiguously demonstrated anti-inflammatory and antioxidative effect of GLP-1 analogs [59,72]. This was also suggested during the LIRA-RENAL trial when lower concentration of inflammation marker hsCRP was obtained in liraglutid treated group in comparison with placebo [63]. Although the mechanism of beneficial renal effect of GLP-1 agonists still remains elusive, it is definitely achieved as a combined effect through glucose lowering treatment and extra-glycemic effects.

## 3. SGLT2 Inhibitors in Diabetic Nephropathy

### 3.1. Mechanism of Action

Sodium/glucose cotransporter 2 (SGLT2) inhibitors are orally administrated hypoglycemic drugs with a novel mechanism of action that is useful across a continuum of diabetes regardless of duration of diabetes, baseline Hba1c or concomitant antidiabetic therapy [73,74]. Glucose reabsorbtion takes place in the proximal tubule via the sodium dependent glucose transporters (SGLT), placed on the apical side of the proximal tubule cell through the basolateral Na, K-ATPase pump [75,76]. SGLT2 is expressed almost entirely in the renal proximal tubules, hence selective inhibition of this protein leads to renal glucose excretion and reduction of plasma glucose levels without influencing other metabolic processes [77]. Sodium glucose cotransporter 2 (SGLT2) is the main luminal glucose transporter placed in the S1 and S2 portions of the proximal tubule (PT) while sodium glucose cotransporter 1 (SGLT1) is placed in the S3 portion and supply fewer than 10% of entire luminal glucose transport [78]. SGLT2 on the apical membrane is connected with GLUT2 on the basolateral part and jointly they reabsorb up to 90% of filtered glucose beneath normoglycaemic conditions [79]. Increased renin angiotensin system (RAS) activity with SGLT2 inhibition is explained by the normal volume depletion with this kind of therapy [80], although a tendency for diminished GFR with SGLT2 inhibition is probably due to enlarged afferent tone over tubuloglomerular feedback [81]. Furthermore, maximal renoprotection from glomerular injury, renal fibrosis, and proteinuria was achieved when luseogliflozin (SGLT2 inhibitor) was combined with the ACE inhibitor, lisinopril [82]. At the early stage of kidney impairment, therapies that prevent the RAS activity are as well indicated, but these approaches are not completely beneficial [83]. One of the possible mechanisms of SGLT2 inhibitors is prevention of glucose influx to the kidney proximal tubular cell responsible for development of diabetic nephropathy [84,85,86]. Histological variation detected in the glomerulus is the traditional focal point in diabetic nephropathy, but it has become extensively recognized that the changes detected in tubulointerstitial fibrosis and in the tubulointerstitium correspond more firmly with impairment in renal function [79]. Dapagliflozin, canagliflozin, and empagliflozin are SGLT2i drugs, useful in reducing hyperglycemia and improvement of glycemic control. They are used in mild renal impairment and have combined beneficial effects such as the lowering of body weight and blood pressure (BP) [73]. The pharmacokinetic properties of SGLT2 inhibitors demonstrate an excellent oral bioavailability, a rather long elimination half-life permitting once daily administration, a short accumulation index, no active metabolites and restricted renal excretion [87]. Moreover, these drugs share an insignificant risk of drug–drug interactions [88]. The risk for hypoglycemia is low because inhibition of SGLT2 does not increase the excretion of insulin or impede with gluconeogenesis. Additionally, there are some indications that SGLT2 inhibition improves beta cell function, perhaps by reducing glucotoxicity [89,90]. Loss of excess calories mediated by glucose excretion in urine results in weight loss and can alleviate the weight gain induced by another classes of hypoglycemic agents. In patients with hypertension SGLT2 inhibitors lower blood pressure as well, probably due to glucosuria, subsequent natriuresis, and diuresis [91]. Absorption of dapagliflozin after oral administration is fast, reaching peak plasma concentrations in 1–2 h it. The main organs included in the metabolism of this drug are the liver and kidneys, where inactive metabolites are produced by enzyme uridine diphosphate-glucuronosyltransferase-1A9 (UGT1A9). Clearance of dapagliflozin by renal excretion is not significant, and there are no drug–drug interactions [88]. Dapagliflozin is capable of reducing body weight and fat mass, improving glycemic control and lowering blood pressure [92,93]. Following the treatment of T2DM with 5 or 10 mg dapagliflozin, a higher risk of mild to moderate urinary tract infections was observed, but without a final dose correlatin among UTI and glucosuria [94].Pharmacokinetic properties of canagliflozin include immediate absorption after oral administration, 65% oral bioavailability within a dose range of 50–300 mg, and in dose-dependent manner, high potential as 99% is bounded to plasma proteins, especially albumin, and finally it is metabolized generally to inactive metabolites [88,95]. As opposed to other SGLT2i, canagliflozin is also capable of minor inhibition of SGLT1. Canagliflozin postpones intestinal glucose absorption in addition to increasing UGE, followed by lowering of postprandial glucose and insulin levels [96]. Canagliflozin treatment is also associated with an UTI and symptomatic vulvovaginal adverse events in female patients with T2DM [97]. Empagliflozin is quickly absorbed in single oral doses achieving Cmax after 1.0–2.0 h. Drug-drug interactions among empagliflozin and other oral glucose lowering agents, cardiovascular medications or various other drugs with narrow therapeutic index were not observed [88,98]. In a fasting and postprandial state in T2DM patients, following administration of empagliflozin or dapagliflozin, paradoxical increase in endogenous glucose production was demonstrated [99]. Nevertheless, plasma glucose levels in these patients were reduced by empagliflozin [98]. Ferrannini et al. showed moderate association of a 10 and 25 mg empagliflozin dose and increased incidence of genital infections, but without increase in UTIs incidence [100]. The capacity of SGLT2i to decrease the plasma glucose levels is directly proportional to the glomerular filtration rate (GFR) and is reduced in chronic kidney disease (CKD). Nevertheless, research underway indicates that SGLT2i can contribute to nephroprotection in diabetes independently of glycemic control [73,101,102,103].

### 3.2. Evidence of Nephroprotection In Vitro and in Animal Models

Cultured human proximal renal tubular cells of patients with type 2 diabetes demonstrate noticeably enlarged levels of SGLT2 mRNA and protein and increased glucose transporter activity [104]. Several experiments using human proximal tubular cells (HK2) showed that SGLT2 inhibition reduced the output of inflammatory and fibrotic markers induced by high glucose levels [73]. The above-mentioned in vitro findings suggest that SGLT2 inhibitors can provide nephroprotection in diabetes by blocking glucose influx to proximal tubule cells [105]. In new preclinical trials, nephroprotection with SGLT2 inhibition has been observed after improvement of glycemic control [82,105,106,107,108,109].

Accordingly, the effect of SGLT2 inhibition on early kidney growth, inflammation, and fibrosis was proposed to result from blood glucose lowering [109]. Thus, blood glucose lowering effect could be responsible for SGLT2i inhibition of inflammation, fibrosis and early renal growth [105]. This hypothesis is supported by several recently published studies conducted in animal models. Inhibition of progression of albuminuria along with drop in plasma glucose by <15 mmol/L was observed in male db/db mice treated with dapagliflozin [107] and females treated with tofogliflozin [109]. Furthermore, Lin et al. showed decrease in albuminuria and glomerulosclerosis followed by improvement of hyperglycemia in male db/db mice treated with empagliflozin [108]. Vallon et al. demonstrated reduction of albuminuria, kidney hypertrophy, and markers of inflammation, proportional to glucose lowering effect in the T1DM model of male Akita mice treated with empagliflozin [105]. The effect of SGLT2 inhibition on diabetic nephropathy, autonomous of blood glucose decrease, was investigated in diabetic eNOS knockout mice [110], while further studies in fat Zucker rats have demonstrated that diabetes enhanced RNA expression of SGLT2 and SGLT1 within the kidney [111]. In addition, proximal tubular cells exposed to the urine of diabetic patients have shown an increase in SGLT2 expression [104]. Diversity of agents have been related to the modification in expression of SGLT1 and 2, including HNF1α and SGK1 [76]. Furthermore, exposure of proximal tubular cells to transforming growth factor β (TGFβ), a profibrotic cytokine, led to upregulation of SGLT2 expression [66].

Interleukin-6 (IL-6) and tumor necrosis factor-α (TNF-α) increased SGLT2 expression in cultured kidney cell lines after exposure for 96–120 h [112], while increase in SGLT2 expression has also been achieved through high glucose-induced pathway exceeding protein kinase A (PKA) and protein kinase C (PKC) reliant pathways [113,114]. Some studies have also shown interaction among the sodium glucose cotransporters and the renin–angiotensin–aldosterone model. For example, losartan decreased SGLT2 expression in diabetic rats in regular or high salt nutrition in animal models [115]. In diabetic rats, enhancement of GLUT2 expression and its translocation to the luminal surface of the proximal tubular cells was observed leading to an increase in glucose reabsorption [78]. One of the potential mechanisms involved in nephroprotection could be linked with a decrease in GLUT 9 expression, a major regulator of urate homeostasis [116,117,118]. The expression of certain profibrotic genes is diminished with empagliflozin, in line with the effects of first line anti-diabetic agent, metformin in diabetic rat model. Gallo at al. presented that a threshold of blood glucose lowering can be necessary to accomplished complete renoprotection in diabetes, since empagliflozin influenced some markers of fibrosis but had no effect on albuminuria and glomerular sclerosis. So, Gallo et al. suggested that adequate, and stable blood glucose lowering, perhaps with multiple medications, including higher- and/or multiple daily-dosing of SGLT2 inhibition in combination with a RAS blockade, can be necessary to accomplished maximal nephroprotection in diabetes [83].

### 3.3. Assessment of Nephroprotective Effect of SGLT 2 Inhibitors in Clinical Trials

Presently, short-term studies are available assuring of renal safety with SGLT2i drugs, however there are no long-term data confirming renal benefit [118] SGLT2i decreases albuminuria, the most important renal risk marker in DN [119]. The albuminuria-lowering effects of SGLT2i have been shown in various studies [120,121] but the precise mechanism is still not clear, and it seems to be independent of alterations in eGFR, systolic BP, body weight, or HbA1c [121]. A placebo-controlled study has demonstrated that canagliflozin 100 mg/day diminished albuminuria around 22% [120], likewise empagliflozin 25 mg/day decreased albuminuria roughly 35% relative to placebo in patients with chronic kidney disease and diabetes mellitus type 2 RAS-based drugs such as ACEi or ARBs, and SGLT2i could have complementary but different mechanisms of action, with diverse outcomes to the kidney system and a potential synergistic effect. In a recently published study, the combination of SGLT2i and RAS blockers was associated with additive nephroprotective effect in diabetic nephropathy compared to either medicament alone [106]. Heerspink et al. showed reduction of albuminuria with dapagliflozin 10 mg/day, compared to placebo in patients with hypertension and diabetes already treated with RAS blockers [121].

Decrease of uric acid in serum is another way where SGLT2i may achieve their nephroprotective effect. Elevated levels of uric acid or hyperuricemia, have been demonstrated to hugely correlate with the possibility of renal impairment in diabetes [122,123,124], and are responsible for microvascular complications in diabetes [125,126]. The beneficial effect of uric acid reduction in serum with SGLT2i can be clinically significant and has been shown in several studies [127,128,129]. The EMPA-REG outcome study has shown that empagliflozin therapy was associated with improvement in all renal function parameters in patients with estimated glomerular filtration rate of at least 30 mL per minute. Empagliflozin significantly decreased worsening or incident nephropathy, request for renal transplantation or dialysis and doubling of serum creatinine levels compared to placebo, while further analysis showed reduction in albuminuria [67].

In the DECLARE trial there was a 24% reduction with dapagliflozin in a composite renal outcome of a ≥40% decrease in estimated glomerular filtration rate (eGFR) (to <60 mL/min/1.73 m^2^), end-stage renal disease (ESRD), or death from renal or CV causes compared with placebo [130]. Included patients had eGFR of at least 60 mL/min at baseline, emphasizing the potential role of dapagliflozin not only in treatment but in the prevention of diabetic nephropathy [131,132]. The CANVAS trial showed a 27% reduction in progression of albuminuria, with a 40% reduction in eGFR, need for renal-replacement therapy, or death from renal causes associated with the use of canagliflozin [131]. The CREDENCE study was the first large-scale outcome trial of an SGLT2 inhibitor canagliflozin with primary kidney outcome defined as doubling of serum creatinine, end-stage kidney disease, or death due to cardiovascular or kidney disease. Almost all patients included in the CREDENCE trial (99%) were treated with ACE inhibitors or ARBs compared to other trials (80%) [133], and had eGFR of 30 to <90 mL per minute as shown in Table 2. The trial was stopped early after a planned interim analysis based on positive results since the relative risk of the renal-specific composite outcome was lower by 34% and the relative risk of end-stage kidney disease was lower by 32%. These data demonstrate that renoprotection was accomplished via the whole spectrum of eGFR levels once again establishing nephroprotecitve effect irrispective of baseline kidney function. The nephroprotective effects of SGLT2i could also benefit cardiovascular outcomes by triggering neurohormonal activation and volume wasting [134,135]. Recent meta-analysis demonstrated that SGLT2 inhibitors reduced the risk of dialysis, transplantation, or death due to kidney disease in individuals with type 2 diabetes and provided protection against acute kidney injury, adding additional evidence endorsing SGLT2i therapy as a corner stone of nephroprotection in diabetics [133]. SGLT2i are not presently recommended in patients with an eGFR lower than 45 mL/min per 1.73 m^2^, to a large degree because of deficient glycemic effectiveness [136,137]. Proof of renoprotection from the above mentioned trials, due to these limitations, is questionable [138].

## 4. Implications of Potential Synergism of GLP 1 RA and SGLT2 Inhibitors on Prevention of Kidney Disease

**Figure 1:**
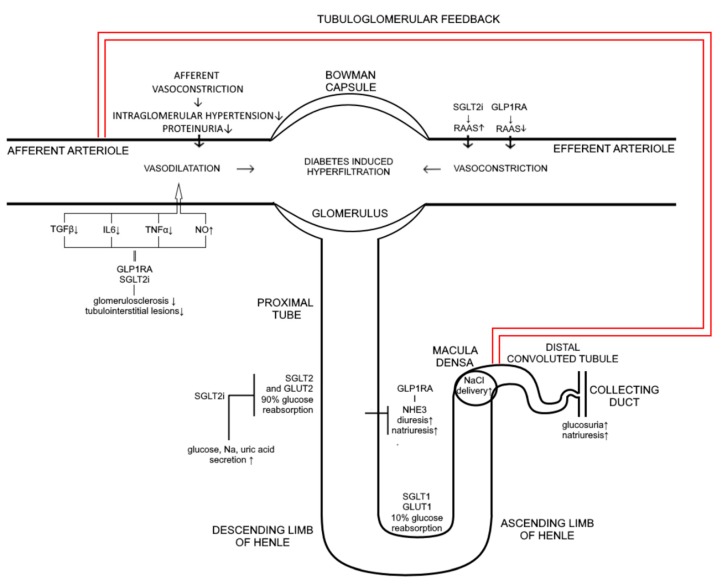
Mechanism of action of SGLT2i and GLP 1RA on kidney. SGLT2i inhibit glucose and sodium transport via SGLT2 and GLUT 2 transporters which are responsible for 90% of glucose reabsorption thus inducing glucosuria, diuresis, natriuresis, and uric acid excretion. At the same time GLP 1RA via NHE3 are also promoting diuresis and natriuresis. Influx of sodium to macula densa is increased thus affecting afferent vasoconstriction causing reduction in intraglomerular pressure and proteinuria through tubuloglomerular feedback. Both, GLP 1RA and SGLT2i induce suppression of inflammatory markers such as TGFβ, IL6, TNFα, decreasing glomerusclerosis and tubulointerstitial lesions and causing afferent vasoconstriction (induction of NO is also involved). GLP 1RA therapy leads to a decrease in RAAS activity causing efferent vasodilatation, while the effect of SGLT2i is quite the opposite, it increases RAAS activity due to natriuresis and volume depletion implying that positive effect on intraglomerular pressure is mediated completely through tubuloglomerular feedback; ↑ increase; ↓ decrease.

Although glycemic control along with blood pressure control and blockade of renin–angiotensin–aldosterone system represents a cornerstone of the prevention of new-onset diabetic nephropathy, 30% of diabetic patients will develop significant renal insufficiency [102,139]. Therefore, novel therapeutic measures as well as diabetic polytherapy are a necessity in prevention and treatment of diabetes mellitus associated complications. Recent studies clearly demonstrate the renoprotective effects of GLP-1 receptor agonists primarily achieved through reduction of new onset microalbuminuria [140]. Although the molecular mechanisms of these beneficial effects are not fully clarified some of renal effects of these drugs have been emphasized. GLP1 receptors present in multiple renal cells are responsible for increased glomerular filtration rate, vasodilatation of the afferent arteriole, and enhanced natriuresis [62]. The state of hyperglycaemia causes enhanced reabsorption of Na+ in proximal tubule and reduced delivery of Na+ to the macula densa with consequent vasodilatation of afferent arteriole, glomerular hypertension, and hyperfiltration [4,141]. Both GLP 1 receptor agonists and SGLT2I promote natriuresis within the proximal tubule acting at different sites. Inhibition of SGLT2 co-transporter beside enhanced glucosuria results in increased natiuresis while GLP-1 receptor agonists promote natriuresis through inhibition of NHE3 (sodium hydrogen exchanger-3) transporter in proximale tubule. Increased delivery of Na+ to macula densa due to inhibition of SGLT2 triggers tubuloglomerlar feedback resulting in afferent vasoconstriction, reduced glomerular pressure and a 30–50% decrease in albuminuria [142]. Despite the natriuretic effect, mechanistic studies and clinical trials failed to exhibit renal hemodynamic vasoconstriction in response to GLP-1 RA treatment resulting in overall neutral GFR effect. However, GLP-1 RA treatment of diabetic patients undoubtedly reduces albuminuria probably due to suppression of inflammation-related pathways [139]. Combination of these antihyperglycemic agents characterizes complementary physiological effect on natriuresis while albuminuria reduction is achieved by different mechanisms. Taken together, potential synergistic and/or additive effects of SGLT2I and GLP-1 receptor agonists at renal function implicate direct beneficial impact on progression of diabetic kidney disease (Figure 1.). In addition, renal benefits could be achieved through common extrarenal effects such as weight reduction and lowering of blood pressure. However, to determine whether a synergistic or additive effect is in question, or perhaps a combination of the two, further studies in vitro and in vivo are needed comparing renal molecular mechanisms and clinical outcomes of combined therapy to each monocomponent separately.

## 5. Conclusions

Both GLP 1 RA and SGLT2i may exhibit direct renoprotective effects through the suppression of inflammatory responses, inhibition of oxidative injury, and prevention of apoptosis as a result of the combined impact of glucose lowering treatment and extra-glycemic effects. Definitely, more intensive glycemic control achieved by addition of GLP-1 agonist and SGLT2i to background antidiabetic therapy justifies a potential benefit on renal function in diabetic patients, however it is obvious that other renoprotective mechanisms exist such as hemodynamic effects, blood pressure control, and body weight loss. Recently, based on the evidence from the trials mentioned above, new European Society of Cardiology (ESC) guidelines in association with European Association for the Study of Diabetes (EASD) position these drugs as the first therapy of choice in patients with diabetes and high cardiovascular risk overthrowing the long-standing paradigm of metformin as the first line therapy in DMT2 [143]. Given the importance of cardiovascular and renal risk reduction in diabetic patients achieved with those therapeutic options, it is only a matter of time before these two classes of drugs will become the gold standard in the treatment of type 2 diabetes.

## Figures and Tables

**Table 1:** The pharmacokinetic properties and renal outcomes of clinical trials with GLP-1 receptor agonists.

Drug.	Dose	Half Life (h)	Elimination	Clinical Study	Renal Benefit
Short-acting GLP-1 receptor agonists					
Exenatide	5–10 μg twice-daily s.c.	2.4	Mostly renal	/	/
Lixisenatide	10–20 μg once-daily s.c.	3.0	Mostly renal	ELIXA [65]	Lower rate of increase in urinary albumin-to-creatinine ratio
Long-acting GLP-1 receptor agonists					
Exenatide	2 mg QW s.c.	2.4	Mostly renal		
Liraglutide	0.6 mg, 1.2 mg or 1.8 mg once-daily s.c.	11.6–13.0	Peptidases and renal 6%; feces 5%	LEADER [64]	↓Nephropathy, ↓UACR, ↓RAS hormone, ↓Progression to macroalbuminuria, ↓Doubling of serum creatinine levels, ↓eGFR of ≤45 mL/min per 1.73 m^2^, ↓The initiation of renal-replacement therapy, ↓Risk of end-stage renal disease or renal death, ↓Plasma renin concentration, renin activity, angiotensin II
Semaglutide	0.5–1.0 mg once-weekly s.c.	165.0–184.0	Peptidases and renal	SUSTAIN-6 [67]	↓Nephropathy ˃35%, ↓Progression to macroalbuminuria, ↓Doubling of serum creatinine levels, ↓eGFR of ≤45 mL/min per 1.73 m^2^, ↓The initiation of renal-replacement therapy
Dulaglutide	0.75–1.5 mg once-weekly s.c.	~112.8	Peptidases and renal	AWARD VII [66]	Reduced albuminuria, slower decline in renal function
Albiglutide	30–50 mg once-weekly s.c.	~120.0	Peptidases and renal	/	/

Abbrevations: s.c., subcutaneous injection; eGFR, estimated glomerular filtration rate in mL/min/1.73m^2^; UACR, urine albumin/creatinine ratio; RAS, renin-angiotensin system; CVR, cardiovascular risk, ↓ decline.

**Table 2:** The pharmacokinetic properties and renal outcomes of clinical trials with SGLT2i.

Drug	Dose (mg)	Half Life (h)	Administration	Clinical Study/Outcome	Renal Benefit
Empagliflozin	10	11.9	Per os, once daily	EMPA-REG OUTCOME [67]/incident or worsening nephropathy and incident albuminuria	↓Nephropathy 39%, ↓Progression to macroalbuminuria, ↓Doubling of serum creatinine levels, ↓the initiation of renal-replacement therapy
Dapagliflozin	10	12.9	Per os, once daily	DECLARE [137]/beneficial effects defined by eGFR status and the attendance or absence of Atherosclerotic cardiovascular illness at baseline	↓eGFR of 40% or more to an eGFR of fewer than 60 mL/min per 1.73 m^2^, ↓Combined risk of end-stage renal disease or renal death, ↓Early prevention and decrease in progression of chronic renal disease in patients with T2DM, 31% reduction in the risk of acute renal injury in the dapagliflozin group compared to placebo group
Canagliflozin	100	over 12	Per os, once daily	CANVAS [67,133]/incident albuminuria, incident of renal failure	↓acute ↓acute kidney injury ↓albuminuria ↓eGFR of 40% ↓the initiation of renal-replacementtherapy ↓death from renal causes kidney injury ↓albuminuria ↓eGFR of 40% ↓the initiation of renal-replacementtherapy ↓death from renal causes
				CREDENCE * [133]/patients with established CKD, incident albuminuria, composite of dialysis, transplantation or death due to renal disease	↓Acute kidney injury, Albuminuria, ↓eGFR of 40%, ↓The initiation of renal-replacement therapy, ↓Death from renal causes in acute kidney injury, ↓Doubling of serum creatinine levels ↓Risk of dialysis and transplantation, ↓Risk of end-stage renal, disease or renal death

Abbreviations: eGFR, estimated glomerular filtration rate in mL/min/1.73m^2^, * fewer than 60 mL/min per 1.73m^2^, * use of RASi was obligatory to entry into the CREDENCE trial; DKD, diabetic kidney disease, ↓ decline.

## Abbreviations

SGLT2i	sodium glucose lowering transporter2 inhibitors
GLP1 RA	glucagon like peptide 1 receptor agonist
GLUT 2	glucose transporter 2
NHE3	sodium-hydrogen exchanger isoform 3
TGFβ	transforming growth factor β
IL6	interleukin 6
TNFα	transforming nuclear factor α
RAAS	renin–angiotensin–aldosterone system
